# Inverse association between Paleolithic Diet Fraction and mortality and incidence of cardiometabolic disease in the prospective Malmö Diet and Cancer Study

**DOI:** 10.1007/s00394-023-03279-6

**Published:** 2023-12-11

**Authors:** Björn Rydhög, Pedro Carrera-Bastos, Yvonne Granfeldt, Kristina Sundquist, Emily Sonestedt, Peter M. Nilsson, Tommy Jönsson

**Affiliations:** 1 Center for Primary Health Care Research, Department of Clinical Sciences in Malmö, Skåne University Hospital, Lund University, Jan Waldenströms Gata 35, CRC, Hus 28 Plan 11, 205 02 Malmö, Sweden; 2 Department of Food Technology, Engineering and Nutrition, Lund University, Lund, Sweden; 3 Nutritional Epidemiology, Department of Clinical Sciences Malmö, Lund University, Lund, Sweden; 4 Internal Medicine–Epidemiology, Department of Clinical Sciences Malmö, Lund University, Lund, Sweden

**Keywords:** Cardiometabolic disease, Cohort study, Diet, HR hazard ratio, ICD International Classification of Diseases, Mortality, Paleolithic Diet Fraction

## Abstract

**Purpose:**

Paleolithic Diet Fraction (PDF) estimates how large a portion of the absolute dietary intake stems from food groups included in the Paleolithic diet. In randomized controlled trials higher PDFs have been associated with healthier levels of cardiometabolic risk markers. Our aim was to build upon these findings by examining associations between PDF and mortality and incidence of cardiometabolic disease in the prospective Malmö Diet and Cancer Study.

**Methods:**

PDF was calculated from an interview-based, modified diet history method, and associations were estimated by using multivariable Cox proportional hazards regression. The examined cohort consisted of 24,104 individuals (44–74 years, 63% women) without previous coronary events, diabetes, or stroke at baseline (1992–1996). A total of 10,092 individuals died during a median follow-up of 18 years.

**Results:**

Median PDF was 40% (0–90%). The adjusted hazard ratios (HR) for PDF as a continuous variable (from 0 to 100%) were for risk of death from all causes 0.55 [95% CI 0.45, 0.66], tumor 0.68 [95% CI 0.49, 0.93], cardiovascular 0.55 [95% CI 0.39, 0.78], respiratory 0.44 [95% CI 0.21, 0.90], neurological 0.26 [95% CI 0.11, 0.60], digestive, 0.10 [95% CI 0.03, 0.30], and other diseases 0.64 [95% CI 0.41, 1.00]. The corresponding HR for risk of coronary event was 0.61 [95% 0.43, 0.86], for ischemic stroke it was 0.73 [95% 0.48, 1.09] and for type 2 diabetes it was 0.82 [95% 0.61, 1.10].

**Conclusion:**

Observational data suggest an inverse association between PDF and all-cause as well as cause-specific mortality and incidence of cardiometabolic disease.

**Supplementary Information:**

The online version contains supplementary material available at 10.1007/s00394-023-03279-6.

## Background

The Paleolithic diet includes fruits and vegetables, roots and tubers, lean meats, fish, seafood, eggs and nuts, and excludes cereal grains, dairy products, legumes, refined fats and sugar [1, 2]. A Paleolithic diet has been hypothesized to be ideal for the prevention of chronic diseases such as coronary heart disease and type 2 diabetes [1]. This hypothesis is supported by the absence of these diseases among recent hunter-gatherer populations and by encouraging results on their risk factors in interventional studies of modern populations [1–4]. Paleolithic Diet Fraction (PDF) estimates how large a portion of the absolute dietary intake stems from food groups included in the Paleolithic diet [5]. In two randomized controlled trials (RCTs) on participants with ischemic heart disease or type 2 diabetes, PDFs were around 80% for the Paleolithic diet and around 40% for a Mediterranean-like and diabetes diet [5, 6]. Higher PDFs were, in these small three-month-long intervention studies, associated with healthier levels of cardiometabolic risk factors such as glycemic control, waist circumference, body weight and blood lipids (total and low-density lipoprotein cholesterol and triglycerides) [5, 6]. Similar associations were found for a similar quantitative measurement called the Paleo Ratio in an RCT of individuals with type 2 diabetes [7]. To expand the clinical significance of these findings for PDF, a study with cardiometabolic disease and mortality outcomes should be performed. Such a study would need to be larger and longer, and therefore more feasible with an observational rather than an interventional study design. For example, a Paleolithic diet pattern score was inversely associated with all-cause and cause-specific mortality in the observational REGARDS (REasons for Geographic and Racial Differences in Stroke) study—a prospective cohort study of 21,423 black and white men and women from all contiguous 48 US states aged ≥ 45 years [8]. The accompanying large number of participants in such studies usually also requires the use of food frequency questionnaires to estimate food intake. Food frequency questionnaires only enable the food intake estimates to be relative. Diet pattern scores such as the Paleolithic diet pattern score in the REGARDS study are therefore based on relative estimates of food intake. The drawback with scores based on relative estimates of food intake is that scores from different populations are not comparable. A relatively high food intake in one population could be a relatively low food intake in another, thus producing very different scores for the same absolute food intake. PDF is based on absolute estimates of food intake and cannot be calculated in studies with relative estimates of food intake [5]. However, in the observational Malmö Diet and Cancer Study (MDCS), the dietary method used enables absolute estimates of food intake.

Therefore, our aim was to build upon previous findings for PDF by examining associations between PDF and mortality and incidence of cardiometabolic disease in the MDCS.

## Methods

### Study population and data collection

The MDCS is a population-based prospective cohort study conducted in Malmö, a city in the south of Sweden. Baseline examinations were conducted between 1991 and 1996. All women born during the period 1923–1950 and all men born 1923–1945, which were living in the city of Malmö, were invited to participate (*n* = 74,138) in the study. Details of the cohort and the recruitment procedures are described elsewhere [9]. The only exclusion criteria were absence of mental capacity and inadequate Swedish language skills (eligible persons = 68,905). The participants filled out questionnaires covering socioeconomic, lifestyle, dietary factors, and recorded meals, and underwent a diet history interview. Anthropometric measurements were conducted by nurses. Weight was measured using a balance-beam scale with subjects wearing light clothing and no shoes. Standing height was measured with a fixed stadiometer calibrated in centimeters. Waist circumference was measured midway between the lowest rib margin and the iliac crest. During the screening period, a total of 28,098 participants (41% of the eligible persons) completed all baseline examinations (Supplemental Fig. 1). Of these, 2129 participants with baseline examinations in 1991 were excluded from the present study since the coding of vegetable and fruit subgroups were handled differently for the 1991 database — compared with later — thus precluding calculation of PDF with legumes excluded from other vegetables as non-Paleolithic [10]. A further 1865 participants with diabetes (*n* = 1177), a history of coronary events (*n* = 507) and/or stroke (*n* = 290) at baseline were also excluded. The remaining 24,104 participants who completed all baseline examinations were included in the present study. All study participants gave written informed consent, and the study was approved by the Regional Ethics Review Board in Lund, Sweden (Dnr LU51-90).

### Dietary data

Dietary data were collected once at baseline. The MDCS used an interview-based, modified diet history method that combined (I) a 7-day food record of meals that varied from day to day, usually lunch and dinner meals, cold beverages, and nutrient supplements, and (II) a 168-item food frequency questionnaire for the assessment of consumption frequencies and portion sizes of regularly eaten foods that were not covered by the 7-day food record. Finally, (III) during a 45-min interview, questions were asked on usual portion sizes and cooking methods of foods recorded during the 7-day food record. The MDCS method is described in detail elsewhere [11, 12]. The PDF association analyses were adjusted for a binary variable called “diet method version” because slightly altered coding routines of dietary data were introduced in September 1994 to shorten the interview time (from 60 to 45 min). The altered coding routines resulted in two slightly different method versions (before or after September 1994) without any major influence on the ranking of individuals [12]. The validity of the MDCS method was evaluated in the Malmö Food study 1984–1985, comparing the method with 18 days of weighed food records [13, 14]. The Pearson correlation coefficients, adjusted for total energy intake, between the reference method and the MDCS method, were in women and men, respectively, for intakes of bread 0.58/0.50, cereals 0.73/0.74, fruits 0.77/0.60, vegetables 0.53/0.65, low-fat milk 0.92/0.90, high-fat milk 0.75/0.76, cheese 0.59/0.47, fish 0.70/0.35, low-fat meat 0.51/0.43 and high-fat meat 0.80/0.40 [13].

The mean daily intake of foods was calculated based on frequency and portion size estimates from the questionnaire and food record. The food intake was converted to energy and nutrient intakes using the MDCS nutrient database where the majority of the nutrient information comes from PC-KOST2-93 from the National Food Agency in Uppsala, Sweden. Energy intake was divided into four categories based on age and sex-specific reference values from the Nordic Nutritional Recommendations (NNR2012) for energy intake at low, medium, and high physical activity level (< low, low-medium, medium–high, > high) [15]. Evaluations of energy and nutrient intakes were based on the Nordic Nutritional Recommendations (NNR2012) [15]. The food intakes in MDCS were aggregated into 33 groups to obtain food groups more frequently consumed in the population, but to keep characteristics related to both dietary behaviors and nutrient content.

Briefly, as previously described, Paleolithic diet is specified on a basis of included and excluded food groups. It includes fruits and vegetables, roots and tubers, lean meats, eggs and nuts but excludes grains, dairy products, legumes, refined fats and sugar [5]. The PDF is calculated as the mean daily fraction between the summed intake of all food belonging to the Paleolithic food groups and the summed intake of all food [5]. For the present study, non-energy-containing beverages such as water, coffee and tea were excluded, and the 33 MDCS food groups were further aggregated into food groups comparable to previous RCTs with PDF (Supplemental Table S1). Among these food groups, which were deemed consistent with our previous classification as Paleolithic in calculations of PDF, were ‘vegetables’, ‘fruits’, ‘potatoes’, ‘eggs’, ‘meat’ (pork, beef, lamb, game meat, poultry, and pure offal), ‘fish’, ‘oil rape seed and olive’, ‘nuts’, and ‘wine’ [5, 6] (Supplemental Table S1). The remaining further aggregated food groups were consequently classified as non-Paleolithic, and consisted of ‘legumes’, ‘juice’, ‘meat products’ (offal as a mixed product or spread, and sausage), ‘milk and milk products’, ‘sweet beverages’, ‘cereals with rice’, ‘fat oil and margarine’, ‘bakery sweets’, ‘jam’, ‘sauce soups’, ‘beer’, ‘spirits’, and ‘remainder miscellaneous’ [5, 6] (Supplemental Table S1). PDF was calculated by weight as the fraction of the mean daily summed absolute dietary intake of Paleolithic food groups divided by the mean daily summed absolute dietary intake of all food groups, as described previously [5, 6] (Supplemental Table S1).

### Ascertainment of mortality, diabetes, coronary events, and ischemic stroke

The participants contributed person-time from the date of enrolment until date of diagnosis, death (10,092 participants), migration from Sweden (188 participants), or end of follow-up (December 2019), whichever occurred first. The mean follow-up time was 20 years for type 2 and unknown type diabetes and 21 years for mortality, coronary events, and ischemic stroke event (range 0–28 years for all).

The National Tax Board provided information on vital status and emigration. Cause of death was obtained from the Cause of Death Registry, where International Classification of Diseases (ICD) codes for the underlying main cause of death were registered. The ICD codes from version 9 and 10 used to record main cause of death group for participants were as follows; tumor (ICD9:140–239, ICD10:C,D00-D48), neurological disease (ICD9:320–389, ICD10:G,H), cardiovascular disease (ICD9:390–459, ICD10:I), respiratory disease (ICD9:460–519, ICD10:J), and digestive disease (ICD9:520–579, ICD10:K). Validation of mortality from the cause of death register ICD codes found that cardiovascular mortality was confirmed in 94% of the participants [16].

Participants with at least two HbA1c values above 6.0% with the Swedish Mono-S standardization system (corresponding to 6.9% in the US National Glycohemoglobin Standardization Program and 52 mmol/mol with the International Federation of Clinical Chemistry and Laboratory Medicine (IFCC) units) [17, 18] were categorized as diabetes cases in the Malmö HbA1c Registry. In addition, diabetes cases were identified via four registries from the National Board of Health and Welfare in Sweden: The Swedish National Inpatient Registry, the Swedish Hospital-based Outpatient Care Registry, the Cause of Death Registry, and the Swedish Prescribed Drug Registry. When available from the regional Diabetes 2000 registry of Scania and its successor the All New Diabetics In Scania (ANDIS) registry, type of diabetes was based on the glycemic parameters, treatment/medication, age at diagnosis, glutamic acid decarboxylase antibodies (GADA), C-peptide and body mass index (BMI). Included were cases with type 2 diabetes and unknown type diabetes. The majority of cases with unknown type diabetes cases were assumed to have type 2 diabetes. Excluded were cases with type 1 diabetes, Latent Autoimmune Diabetes in Adults (LADA), pregnancy diabetes, secondary diabetes, and other types of diabetes. If available, we used information on the date of diabetes diagnosis from two registries prioritized in the following order: (a) the Diabetes 2000 registry of Scania and (b) the Swedish National Diabetes Registry. These registries required a physician diagnosis according to established diagnosis criteria (fasting plasma glucose concentration ≥ 7.0 mmol/L or fasting whole blood concentration ≥ 6.1 mmol/L, measured on two different occasions).

Information about prevalent and incident coronary and ischemic stroke events was taken from the national Swedish Hospital Discharge register, Cause-of-death register, and the local stroke register in Malmö (STROMA). A coronary event was defined based on codes 410–414 (fatal or non-fatal myocardial infarction or death due to ischemic heart disease) in the International Classification of Diseases, 9th Revision (ICD-9). Ischemic stroke was defined based on code 434 (ICD-9) and diagnosed when computed tomography, magnetic resonance imaging or autopsy could verify the infarction and/or exclude hemorrhage and non-vascular disease. If neither imaging nor autopsy was performed, the stroke was classified as unspecified. Hemorrhagic and unspecified stroke cases (ICD-9 code 430, 431 and 436) were excluded since these subtypes of stroke do not have the underlying risk factors associated with diet as ischemic stroke has.

### Other variables

Information on age was obtained from the personal identification number, which is assigned to individuals upon birth or permanent immigration to Sweden. BMI was calculated from measurements of weight and height and divided into four categories(< 18.50, 18.50–24.99, 25–29.99, ≥ 30.00 kg/m^2^) [19]. Leisure time physical activity was assessed by asking the participants to estimate the number of minutes per week spent on 17 different activities. The duration was multiplied with an activity-specific intensity coefficient and an overall leisure time physical activity score was created and divided into quintiles [16]. The smoking status of the participants was defined as current smokers (including irregular smokers), ex-smokers, and never-smokers. The calculated mean daily alcohol intake from the 7-day food record was divided into quintiles by sex. Participants were divided into four categories according to their highest level of education (≤ 8, 9–10, 11–13 years, or university degree). Season was defined as the season of diet data collection (winter, spring, summer, and fall/autumn).

### Statistics

The SPSS statistical computer package (version 28.0; IBM Corporation, Armonk, NY, USA) was used for all statistical analyses. Normality was assessed both graphically and numerically including Kolmogorov–Smirnov tests and many variables were non-normally distributed with outliers. Baseline characteristics were examined using Mann–Whitney *U* test, Chi-square test and Spearman rank test. Cox proportional hazards regression model was used to examine associations between the predictor PDF (both continuous and by quintiles) and risk of the outcomes all-cause death, main cause of death group, coronary event, ischemic stroke, incidence of type 2 diabetes, and incidence of either type 2 or unknown type diabetes. PDF as a continuous variable varies between 0 and 1. With PDF as a continuous variable, the hazard ratio indicates the change in the risk if PDF rises by one unit from 0 to 1, thus comparing a PDF of 0% with a PDF of 100%. For every 10% difference in PDF the percentage difference in risk will then be 10 multiplied with the difference between 1 and the hazard ratio. Years of follow-up was used as the underlying time variable. Predictor selection for our Cox regression model was based on our inferential goal of evaluating a predictor of primary interest, namely PDF. In pursuing this goal, relatively inclusive models are more likely to minimize the central problem of confounding. Predictors necessary for face validity as well as those that behave like confounders should be included in the model whilst avoiding predictors that are alternate measures of or mediators of our predictor of primary interest [20]. The full Cox regression model included the following predictors obtained from baseline examination: PDF (continuous or categorical), age (continuous), sex, diet method version, season, leisure time physical activity, smoking, alcohol intake, education, BMI, energy intake, born in Sweden, and living alone (categorical). The first PDF quintile was used as the reference, indicating participants with the lowest PDF. The continuous variables BMI and energy intake were converted into categorical predictors for the Cox regression model due to violation of its linearity assumption. The predictors were identified from the literature and indicated potential confounding in the MDCS cohort due to associations with examined outcomes and dietary intakes. Effect measure modification by predictors was assessed with stratification and interaction terms in Cox regression analyses. Since associations between PDF and examined outcomes could be mediated via the effects of PDF on BMI and energy intake, we also performed Cox regression analyses with BMI and energy intake excluded from the full model. Missing values for the predictors of the full model Cox regression were treated as separate categories. In sensitivity analyses, we excluded participants with missing values for predictors of the full model Cox regression. To assess the proportional hazards assumption, log minus log graphs were used to test interactions between the underlying time variable and examined categorical predictors (age categorized into three age categories 44–54, 55–64 and 65–74 years for this assessment). All statistical tests were two-sided and statistical significance was assumed at *p* < 0.05.

## Results

Baseline participant characteristics are presented in Table 1, median daily food intake at baseline in Table 2, and median daily nutrient intake at baseline in Supplemental Table S2. A higher quintile of PDF was associated with female sex, lower age, shorter height, lower weight, smaller waist, lower blood pressure, less smoking, more exercise, higher education, and not being born in Sweden or living alone (Table 1). Median PDF was 40% and quintile of PDF correlated positively with food intake from all Paleolithic food groups and negatively with food intake from all non-Paleolithic food groups and total food intake by weight and energy (Table 2). Food groups with high intakes by weight correlated the most with quintiles of PDF, with the highest correlations for vegetables and fruit among the Paleolithic food groups, and for milk and milk products among the non-Paleolithic food groups (Table 2). Macronutrient intake by energy percent was overall as recommended but at the lower bound for carbohydrates with quintile of PDF correlating positively with protein, fiber and alcohol, but negatively with fat and carbohydrates (Supplemental Table S2). For all quintiles of PDF, median absolute intakes of dietary fiber were below recommendations and micronutrient intakes were overall as recommended except for too-low intakes of vitamin D, folate, and selenium, as well as too-high intakes of sodium (Supplemental Table S2). The correlations between quintiles of PDF and absolute intakes of micronutrients were approximately equally divided into positive and negative, but the correlations between quintiles of PDF and relative intakes of micronutrients (relative to total food weight) were mostly positive with some neutral and only negative for retinol, riboflavin, and calcium (Supplemental Table S2). Intakes of saturated fat and the sum of alpha-linolenic acid and gamma-linolenic acid were above and below recommendations, respectively, with a negative correlation between PDF and relative intakes of saturated fat (Supplemental Table S2).

**Table 1:** Baseline Characteristics

Variable	(IQR, Min–Max)	Quintile of PDF					*P*^*a*^	*r*_*s*_	Missing values, *n*
		1	2	3	4	5			
PDF, % *Mdn*	40 (15, 0–90)	27	34	40	46	56			0
Age, years *Mdn*	57 (13, 44–74)	58	58	58	57	56	< .001	– .05	0
Height, cm *Mdn*	168 (13, 127–203)	171	169	168	167	166	< .001	– .17	29
Weight, kg *Mdn*	72 (18, 31–170)	74	72	72	71	70	< .001	– .09	30
BMI, kg/m2 *Mdn*	25 (5, 14–51)	25	25	25	25	25	.2	.01	30
Waist, cm *Mdn*	82 (19, 50–152)	86	84	82	80	79	< .001	– .15	41
Hip, cm *Mdn*	97 (11, 49–178)	97	97	97	97	97	.1	– .01	41
SBP, mm Hg *Mdn*	140 (26, 61–230)	140	140	140	140	138	< .001	– .05	29
DBP, mm Hg *Mdn*	85 (10, 40–136)	86	85	85	85	84	< .001	– .04	31
Women, %	63	46	56	63	70	78	< .001		0
Smokers (ex/current), %	62	67	61	60	59	62	< .001		9
Alcohol (highest quintile)^b^, %	20	16	16	19	22	27	< .001		0
LTP (highest quintile), %	20	19	20	20	20	21	.03		119
Education (> 10 years), %	33	29	31	32	34	37	< .001		55
Born in Sweden, %	88	89	90	89	88	84	< .001		9
Living alone, %	25	29	25	24	23	22	< .001		16

*Note.* Participants without prevalent coronary event, stroke or diabetes at baseline examination 1992–1996 (*N* = 24,104) from the Malmö Diet and Cancer Study. *PDF*  Paleolithic Diet Fraction. *SBP and DBP*  Systolic and diastolic blood pressure, *LTP*  Leisure Time Physical activity

^a^Chi-square test across quintiles of PDF or Spearman rank test with quintile of PDF as predictor.^b^By sex

**Table 2:** Median daily food group intake at baseline

Variable	(IQR, Min–Max)	Quintile of PDF					*P*^*a*^	*r*_*s*_
		1	2	3	4	5		
PDF, %	40 (15, 0–90)	27	34	40	46	56		
Total food group weight, g	1691 (623, 330–5386)	1934	1759	1707	1613	1486	< .001	-.31
Total food group energy, MJ	9.1 (3.4, 2.2–35.1)	10.3	9.7	9.3	8.7	7.9	< .001	-.32
Total food group energy per gram, kJ	5.4 (1.3, 2.3–12.7)	5.3	5.5	5.4	5.4	5.4	.7	.00
Paleolithic food groups, g	667 (289, 0–4089)	495	604	680	743	848	< .001	.55
Vegetables, g	145 (110, 0–1177)	98	128	151	168	201	< .001	.42
Fruits, g	167 (152, 0–2782)	107	146	175	203	249	< .001	.42
Potatoes, g	109 (84, 0–1447)	103	111	113	112	107	< .001	.03
Eggs, g	19 (22, 0–245)	18	19	20	20	19	< .001	.02
Meat^b^, g	94 (60, 0–593)	85	91	96	96	99	< .001	.09
Fish, g	39 (41, 0–527)	31	36	41	43	47	< .001	.17
Oil rape seed and olive, g	0 (0, 0–74)	0	0	0	0	0	< .001	.09
Nuts, g	0 (2, 0–147)	0	0	0	0	0	< .001	.06
Wine, g	21 (71, 0–1127)	0	0	29	43	54	< .001	.30
Non-Paleolithic food groups, g	1001 (515, 75–4432)	1431	1157	1023	866	641	< .001	-.70
Legumes, g	0 (15, 0–520)	0	0	0	0	0	< .001	-.09
Juice, g	1 (100, 0–1500)	1	2	4	1	0	< .001	-.10
Meat products^c^, g	24 (35, 0–390)	33	28	25	20	15	< .001	-.24
Milk and milk products, g	396 (316, 0–3339)	585	471	413	351	238	< .001	-.47
Sweet beverages, g	8 (94, 0–3000)	63	36	21	0	0	< .001	-.30
Cereal grains with rice, g	132 (82, 0–1114)	157	147	135	124	108	< .001	-.27
Fat oil and margarine, g	30 (31, 0–209)	33	34	32	29	25	< .001	-.11
Bakery sweets, g	65 (57, 0–715)	79	73	68	60	50	< .001	-.23
Jam, g	12 (20, 0–218)	15	14	13	11	8	< .001	-.15
Sauce soups, g	3 (10, 0–1006)	3	3	3	2	2	< .001	-.11
Beer, g	83 (193, 0–3286)	119	107	94	71	36	< .001	-.21
Spirits, g	0 (7, 0–550)	0	0	0	0	0	< .001	-.03
Remainder miscellaneous, g	4 (4, 0–692)	4	4	4	4	4	.04	-.01

*Note.* Participants without prevalent coronary event, stroke or diabetes at baseline examination 1992–1996 (*N* = 24,104) from the Malmö Diet and Cancer Study. Non-energy containing beverages excluded. *PDF*  Paleolithic Diet Fraction

^a^Spearman rank test with quintile of PDF as predictor. ^b^Pork, beef, lamb, game meat, poultry, and pure offal. ^c^Offal as a mixed product or spread, and sausage

**Fig. 1:**
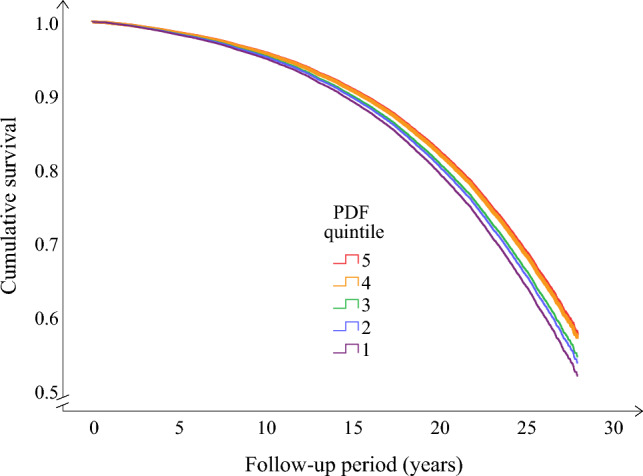
Kaplan–Meier curve of cumulative survival by quintile of PDF among participants (*n* = 24,104) in the Malmö Diet and Cancer cohort (1992–2019)

Main cause of death and incident event outcomes are presented in Table 3. For main cause of death outcomes, a higher PDF was associated with a lower risk of death from all causes (10,092 deaths after a median of 18 years of follow-up), tumor (3,606 deaths, 36%) and cardiovascular (3,108 deaths, 31%), respiratory (711 deaths, 7%), neurological (559 deaths, 6%), and digestive disease (284 deaths, 3%, Table 3; Fig. 1). There was no association between the PDF and the risk of other individual main causes of death groups, but higher PDF was non-significantly associated (*p* = 0.05) with a lower risk of death when those groups were aggregated into one ‘Other disease’ main cause of death group (1824 deaths, 18%, Table 3). Based on the Cox regression results for PDF as a continuous variable, every 10% higher PDF resulted in a 4.5% lower risk of all-cause death (Table 3). For incident event outcomes, a higher PDF was associated with a lower risk of coronary events, with non-significant similar associations for ischemic stroke and type 2 diabetes including unknown type diabetes (*p* = 0.1 and 0.2, respectively, Table 3). The association between PDF and type 2 diabetes excluding unknown type diabetes (1,948 cases during 481,620 person-years) was similar albeit weaker (*p* = 0.3). These associations were unaffected in sensitivity analyses that excluded participants with missing values.

**Table 3:** Hazard ratios for main cause of death and incidence of coronary event, ischemic stroke, and type 2 diabetes

Outcome	Cases/person years	Hazard Ratio [95% CI]		Quintile of PDF					*P*^*b*^
		PDF^a^	*P*^a^	1	2	3	4	5	
*Main Cause of death*									
All-cause	10,092/524,071	0.55 [0.45, 0.66]	< .001	1.00	0.96 [0.90, 1.01]	0.93 [0.87, 0.98]	0.86 [0.81, 0.92]	0.85 [0.79, 0.91]	< .001
Tumor	3,606/524,071	0.68 [0.49, 0.93]	.02	1.00	0.97 [0.88, 1.07]	1.00 [0.91, 1.11]	0.90 [0.81, 1.00]	0.91 [0.82, 1.02]	.05
Cardiovascular disease	3,108/524,071	0.55 [0.39, 0.78]	.001	1.00	0.96 [0.87, 1.07]	0.94 [0.85, 1.05]	0.84 [0.75, 0.94]	0.86 [0.76, 0.97]	.001
Respiratory disease	711/524,071	0.44 [0.21, 0.90]	.03	1.00	0.92 [0.74, 1.14]	0.77 [0.61, 0.97]	0.95 [0.76, 1.19]	0.70 [0.54, 0.91]	.03
Neurological disease	559/524,071	0.26 [0.11, 0.60]	.002	1.00	0.97 [0.76, 1.24]	0.87 [0.67, 1.13]	0.68 [0.52, 0.90]	0.65 [0.49, 0.88]	< .001
Digestive disease	284/524,071	0.10 [0.03, 0.30]	< .001	1.00	0.75 [0.54, 1.05]	0.71 [0.50, 1.00]	0.60 [0.41, 0.88]	0.53 [0.35, 0.79]	.001
Other disease	1,824/524,071	0.64 [0.41, 1.00]	.05	1.00	0.96 [0.83, 1.10]	0.87 [0.75, 1.01]	0.91 [0.79, 1.06]	0.90 [0.77, 1.05]	.1
Coronary event	2,988/506,454	0.61 [0.43, 0.86]	.01	1.00	0.99 [0.89, 1.10]	0.91 [0.81, 1.01]	0.96 [0.86, 1.08]	0.84 [0.74, 0.96]	.01
Ischemic stroke	2,259/506,603	0.73 [0.48, 1.09]	.1	1.00	0.97 [0.86, 1.10]	0.98 [0.86, 1.11]	0.96 [0.84, 1.09]	0.90 [0.78, 1.04]	.2
Type 2 diabetes^c^	3,995/481,620	0.82 [0.61, 1.10]	.2	1.00	0.96 [0.88, 1.06]	1.00 [0.91, 1.11]	0.97 [0.88, 1.07]	0.92 [0.83, 1.02]	.2

*Note.* Cox regression model adjusted for age, body mass index, total energy intake, diet method version, season, leisure time physical activity, smoking, alcohol intake, education and living alone at baseline examination as well as sex and born in Sweden. Participants without prevalent coronary event, stroke or diabetes at baseline examination 1992–1996 (*N* = 24,104) from the Malmö Diet and Cancer Study. *PDF*  Paleolithic Diet Fraction

^a^PDF continous variable between 0 and 1, thus comparing a PDF of 0% with a PDF of 100%. ^b^Trend across categories (numeric values 1–5 of the categorical variable Quintile of PDF are treated as a score). ^c^Type 2 or unknown type diabetes

Missing values were found for leisure time physical activity (*n* = 119), cause of death (*n* = 61), education (*n* = 55), BMI (*n* = 30), living alone (*n* = 16), smoking (*n* = 9), and born in Sweden (*n* = 9), with little overlapping between participants. We did not find any effect measure modification and also no effect mediation by BMI or energy. The proportional hazards assumption was considered to be satisfied for all predictors since their log minus log graphs showed parallel lines without crossing.

## Discussion

PDF was inversely associated with the risk of death from all causes, tumor, cardiovascular, respiratory, neurological, and digestive disease. PDF was also inversely but non-significantly associated with death from other diseases combined. For incident event outcomes, PDF was inversely associated with the risk of coronary events and also inversely, but non-significantly associated, with ischemic stroke and type 2 diabetes. Besides longer follow-up, the present study extends previous research by examining associations for a measure of how Paleolithic a diet is based on absolute food intakes with mortality and incidence of cardiometabolic disease. This enables comparison between studies not possible for Paleolithic diet pattern scores based on relative food intakes.

The inverse association between PDF and risk of death from all causes in this prospective cohort study among 24,104 MDCS participants aged 44–74 years (mean age 57 years (SD 10 years), 63% women) is supported by similar results by Whalen et al*.* (2017) for a Paleolithic diet pattern score based on relative food intakes in the REGARDS study, a prospective cohort study of 21,423 black and white men and women from all contiguous 48 US states aged ≥ 45 years (mean age 66 years (SD 9 years), 56% women) [8].

This Paleolithic diet pattern score was developed by Whalen et al*.* (2014) and assigns a quintile rank (and a corresponding score from 1 to 5) of food intake by sex for each of 14 score components [21]. Higher points were given for a higher intake of foods considered characteristic of a Paleolithic diet pattern; vegetables, fruits, lean meats, fish, nuts, fruit and vegetable diversity, and calcium, or lower intake of foods considered uncharacteristic of a Paleolithic diet pattern; processed and non-lean red meats, sodium, dairy, grain and starches, baked goods, sugar-sweetened beverages, and alcohol [21]. Higher points thus indicate a food intake more in line with a Paleolithic diet pattern relative to the examined population [21]. Further support comes from another research group in the observational Moli-sani Study, a prospective cohort study of 22,849 men and women in Italy aged ≥ 35 years (mean age 55 years (SD 12 years), 52% women), which used the same Paleolithic diet pattern score developed by Whalen et al*.* (2014) to find a similar inverse association with risk of death from all causes [22]. The same Paleolithic diet pattern score, although now re-termed evolutionary-concordance diet pattern score due to perceived constraints in investigating the Paleolithic diet in the modern context, was also used to find a similar albeit non-significant inverse association with risk of death from all causes in the observational Iowa Women’s Health Study, a prospective cohort study of 41,836 women in Iowa, USA, aged 55–69 years (mean age 62 years; SD 4 years) [23]. At baseline, the MDCS, IWHS, Moli-sani Study and REGARDS all exhibited a moderate agreement with the Mediterranean dietary pattern [24–27], with mean daily dietary energy intakes of 9, 8, 9 and 7 MJ, respectively (Table 2) [8, 28, 29]. In the same studies the average BMI was 25, 27, 28 and 29 kg/m^2^ (Table 1) [29–31], the proportion of current smokers was 34%, 15%, 25% and 14% [30, 32–34], and the mean daily alcohol intake was 7, 4, 16, and 13 g, respectively (Supplemental Table S2) [8, 23, 32].

The support from observational studies using Paleolithic diet pattern scores for an inverse association between PDF and risk of death from tumor is not as consistent, with similar associations found in some studies for risk of death from all-cancer and diagnosis of breast cancer and colorectal adenoma [8, 21, 35–37], but also with no association found in some studies for risk of death from all-cancer and diagnosis of colorectal cancer [22, 23, 31]. The weaker support is consistent with the association between PDF and risk of death from a tumor also being the weakest of the associations found for the main cause of death outcomes. Additional support can possibly come from an inverse association with biomarkers of chronic systemic inflammation and oxidative stress found in an observational study in Paleolithic diet pattern score, since both markers have been associated with cancer, but also with other chronic diseases such as cardiovascular disease [38].

The support from observational studies using Paleolithic diet pattern scores for an inverse association between PDF and risk of death from cardiovascular disease is also not consistent, with similar associations found in some studies [8, 39], but no association found in other studies [22, 23, 28, 40]. However, more support for this latter association comes instead from within this study, in the form of a congruent inverse association between PDF and risk of coronary events, and with a similar, albeit non-significant association, for ischemic stroke and type 2 diabetes. Similar support also comes from an inverse association with the risk of type 2 diabetes and hypertension found in an observational study of a Paleolithic diet pattern score [41]. Furthermore, the beneficial association between PDF and baseline cardiovascular risk factors in the current study, together with beneficial associations between the Paleolithic diet, PDF and similar quantitative measurement and cardiometabolic risk factors in previous intervention studies, also concur with an inverse association between PDF and risk of death from cardiovascular disease [4–7, 42, 43]. In a recent network meta-analysis of the effects of dietary patterns on cardiometabolic risk factors in RCTs, the Paleolithic diet received the highest all-outcomes-combined average surface under the cumulative ranking curve (67%), followed by Dietary Approaches to Stop Hypertension, DASH (62%) and Mediterranean diets (57%), whereas western habitual diet was lowest (36%) [43].

The inverse association between PDF and the risk of death from respiratory diseases was unexpected and, to the best of our knowledge, no study has investigated this association. However, for the inverse association between PDF and risk of death from neurological diseases, limited support comes from intervention studies where the Paleolithic diet — sometimes modified — has increased cognitive function, hippocampal volume, and serum brain-derived neurotrophic factor levels, and reduced fatigue and increased quality of life, exercise capacity, and hand and leg function in patients with multiple sclerosis [44–46].

The inverse association between PDF and the risk of death from digestive diseases was based on fewer cases and should be interpreted with caution. However, some support for this association comes from improved endoscopic inflammation and symptoms in an intervention study on the autoimmune protocol diet, which is an extension of the Paleolithic diet, in patients with inflammatory bowel disease [47]. Less clear support comes from observational studies of gut microbiota, with higher microbial richness and biodiversity among the Hadza hunter-gatherers compared to Italian controls [48], as well as higher biodiversity among Italians adhering to a Paleolithic diet compared to Italians adhering to a Mediterranean diet [49].

In this cohort, the median PDF was 40%, which is similar to the PDFs found for the Mediterranean-like and diabetes diet in our previous intervention studies [5, 6]. Median PDF in the lowest PDF quintile of this cohort was 27%, which is similar to an estimate of a western habitual diet [50], and therefore indicates that the MDCS cohort as a whole ate more Paleolithic foods than a typical western population. Median PDF in the highest quintile of PDF of this cohort was 56%, which is quite far from the PDF of around 80% found for the Paleolithic diet in our intervention studies [5, 6]. A similar quantitative measurement called the Paleo Ratio was even higher at 94% for the Paleolithic diet in another intervention study; this was possibly due to greater counselling efforts [7]. In this study, based on the Cox regression results for PDF as a continuous variable, every 10% higher PDF results in a 4.5% lower risk of all-cause death.

Regarding PDF and nutrient intake in the MDCS, macronutrient intake by energy percent was overall as recommended but at the lower bound for carbohydrates with PDF correlating positively with protein, fiber and alcohol, but negatively with fat and carbohydrates. This is in line with previous studies showing that the Paleolithic diet is generally high in protein and low in carbohydrates [50–52]. Most relative micronutrient intakes were positively associated with PDF but calcium intake was negatively associated, which is also in line with previous studies on the Paleolithic diet [50, 51].

### Strengths

A strength of this study is its large sample size and long follow-up period of 20 years on average. The MDCS dietary assessment method measures both habitual and recent intakes, and the documented relative validity and reproducibility of food intake indicates high-quality dietary data. Moreover, it is a population-based prospective study, which reduces the risk of selection bias and reverse causation. Further strengths are the extensive information on potential confounders, and that diet was measured with a modified diet history method including a 7-day food record for cooked meals.

### Limitations

A major limitation of this observational study is that diet and other lifestyle factors were only measured at baseline, precluding the possibility of studying and adjusting for changes over time. However, reproducibility studies including similarly aged participants show acceptable agreement between repeated dietary measurements [53, 54], possibly because dietary habits are often already established at younger ages. For other lifestyle factors, the impact of this limitation can be expected to vary. For BMI, both decreases and increases over time are associated with greater risk of cardiovascular outcomes [55] indicating that the risk that is based on a single measurement at baseline could be underestimated. Changes in leisure-time physical activity are inversely associated with changes in cardiovascular outcomes [56]. Leisure-time physical activity in Sweden have increased during the time period of this study which indicates that the associated risk from a single measurement at baseline could be overestimated [57, 58]. Decreased smoking prevalence and increased alcohol intake among the general population in Sweden during the time period of this study indicate that the associated risk from a single measurement at baseline could be overestimated and underestimated, respectively [59, 60].

Another limitation is that the study sample only consisted of Swedish individuals aged 44–74 years and mostly women (63%) from an urban setting, which decreases generalizability to other age groups, men, and rural settings. Furthermore, residual confounding cannot completely be ruled out despite adjustment for several confounders, and changes over time in recognition, diagnosis, certification and/or coding of different diseases may have affected the registered death causes and consequently also the found associations. A reflection of this may be the inconsistent trends in hazard ratios across quintiles of PDF seen for several cause-specific mortality outcomes, requiring greater caution in their interpretation. In addition, since one of our main objectives was to examine all-cause mortality, we did not take competing risks into account in our statistical analysis, hence why we cannot exclude that this may be a concern regarding the results on cause-specific mortality. Finally, a limitation of this study is that other dietary estimates and pattern scores were not calculated, precluding the possibility of direct comparison with PDF. Such comparisons, albeit not within the planned scope of this study, would be interesting and are considered for future studies.

### Future research

The results suggest the clinical importance of PDF which warrants further study. Future dietary research should assess PDF whenever possible to benefit from the comparability of PDF between studies.

## Conclusion

Observational data confirm and expand our findings in previous RCTs and suggest an inverse association between PDF and all-cause as well as cause-specific mortality, and incidence of cardiometabolic disease. The findings underline the importance of considering PDF in dietary research and future dietary guidelines and policies.

### Supplementary Information

Below is the link to the electronic supplementary material.

Supplementary file1 (PDF 201 KB)

Supplementary file2 (PDF 255 KB)

Supplementary file3 (PDF 187 KB)

## Data Availability

Data described in the manuscript can be made available upon request pending application and approval by the chair of the steering committee for the cohort.

## Abbreviations

PDF: Paleolithic Diet Fraction
RCTs: Randomized controlled trials
MDCS: Malmö Diet and Cancer Study
HR: Hazard ratio
ICD: International Classification of Diseases
REGARDS: REasons for Geographic and Racial Differences in Stroke
HbA1c: Glycated hemoglobin
BMI: Body mass index
SBP and DBP: Systolic and diastolic blood pressure
LTP: Leisure time physical activity
