# Supplementary figures and images for: Inverse association between Paleolithic Diet Fraction and mortality and incidence of cardiometabolic disease in the prospective Malmö Diet and Cancer Study
Source: Eur J Nutr. 2023 Dec 11;63(2):501–12. doi: 10.1007/s00394-023-03279-6 (PMC10899283; doi:10.1007/s00394-023-03279-6)

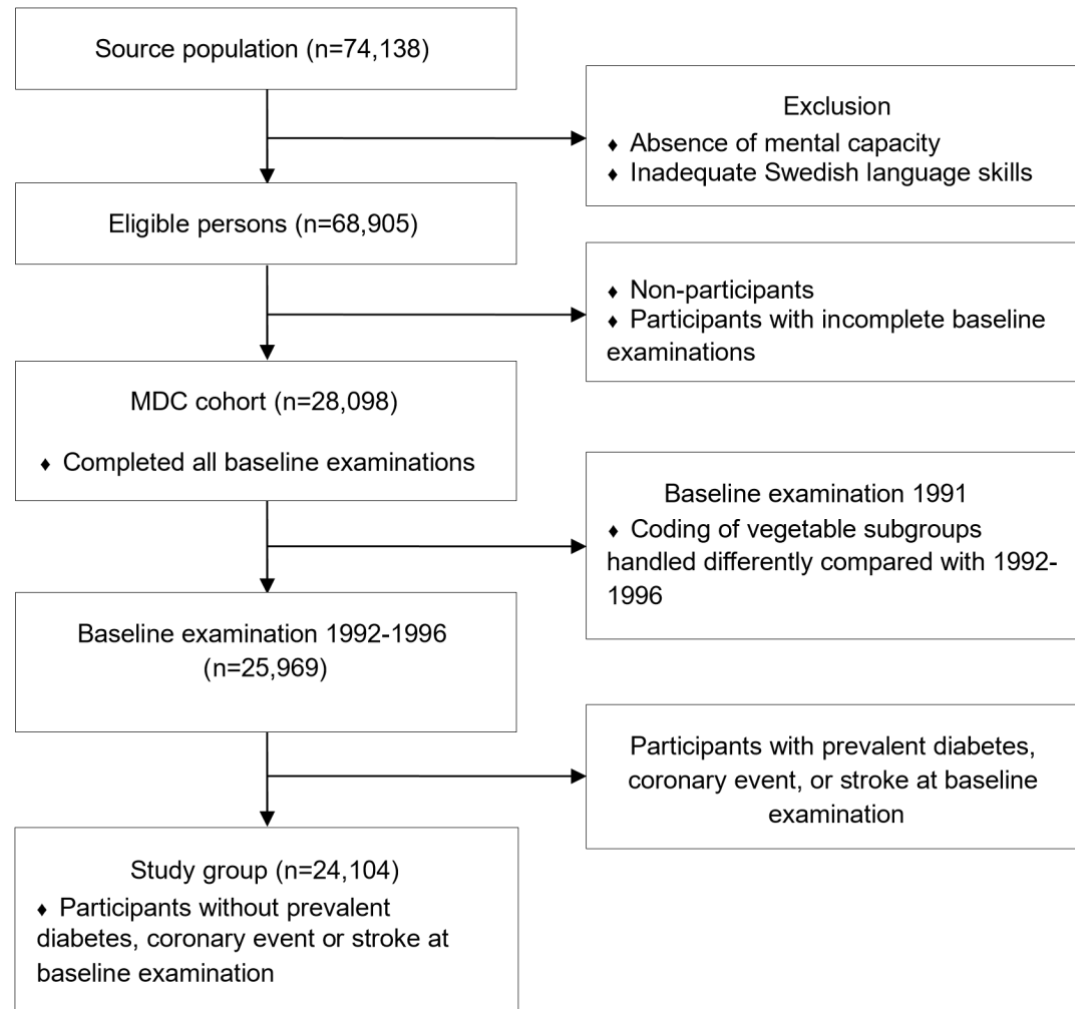

**Supplemental Figure 1.** A flow chart of sample selection from the Malmö diet and Cancer study

Supplement: Supplementary file 1 — Supplementary file1 (PDF 201 KB) [file 394_2023_3279_MOESM1_ESM.pdf]
