# Supplementary material for: Inverse association between Paleolithic Diet Fraction and mortality and incidence of cardiometabolic disease in the prospective Malmö Diet and Cancer Study
Source: Eur J Nutr. 2023 Dec 11;63(2):501–12. doi: 10.1007/s00394-023-03279-6 (PMC10899283; doi:10.1007/s00394-023-03279-6)
Supplement: Supplementary file 2 — Supplementary file2 (PDF 255 KB) [file 394_2023_3279_MOESM2_ESM.pdf]

Supplemental Table S1

Construction of Paleolithic Diet Fraction

Paleolithic Diet Fraction (PDF) calculation	Paleolithic and non-Paleolithic food groups aggregated from MDCS food groups	Aggregated MDCS food groups briefly described	Aggregated MDCS food group variable names (full descriptions in Wirfält E, Sonestedt E (2016) The modified diet history methodology of the Malmö Diet and Cancer cohort)
PDF was calculated by weight as the fraction of the mean daily summed absolute dietary intake of Paleolithic food groups divided by the mean daily summed absolute dietary intake of all food groups	Paleolithic food groups		
	Vegetables	Vegetables (not legumes)	g2vegthf-glegumh-glegumf
	Fruits	Fruits, berries	g2ncitrt+g2citrt
	Potatoes	Potatoes	gpotboil+gpotrec+gpotfri+gpotdfri
	Eggs	Eggs	geggt
	Meat	Pork, beef, lamb, game meat, poultry, pure offal	gporklf+gporkmf+gporkhf+gporks+gbeeflf+gbeefhf+gbeefs+glamblf+glambhf+ggamet+gpoultry
	Fish	Fish, shellfish, molluscs	gfishlf+gfishhf+gfishm+gfihelf+gfihehf+gfishs+gshellft
	Oil rape seed and olive	Rape seed and olive oil	goil2+goil4
	Nuts	Nuts, seeds, almond paste	gnuts
	Wine	Wine	gwine
	Non-Paleolithic food groups		
	Legumes	Legumes	glegumh+glegumf
	Juice	Juice from vegetables and fruits	gcarrju+gvegju+gcitrju+gncitrju
	Meat products	Offal as a mixed product or spread, sausage	goffalm+goffal+goffals+gsaust+gsauss
	Milk and milk products	Milk, cream, cheese, butter, milk fat-based margarine	gmilklf+gmilkmf+gmilkhf+gmilkylf+gmilkymf+gmilkyhf+gcreamlf+gcreammf+gcreamhf+gcheeslf+gcheesmf+gcheeshf+gbutter+gmargd1+gmargd2
	Sweet beverages	Soft drinks	gsofdr

Cereal grains with rice	Cereal grains, rice	gcelfin+gcelfls+gcelfhs+gcehfn+gcehfls+gcehfhs+gflourt+gbrelfi+gbremfi+gbrehfi+gbrexhfi+gcrbrlfi+gcrbrmfi+gcrbrhfi+gbirulei+gbiruhfi+gripat+ggruel
Fat oil and margarine	Oil (not rape seed and olive), margarine (not milk fat-based), mayonnaise/dressing	goil1+goil3+gmarghf1+gmarghf2+gmarghf3+gmarghf4+gmarghf5+gmargmf+gmarglf1+gmarglf2+gmarglf3+gmarglf4+gdresslf+gdressmf+gdresshf
Bakery sweets	Cakes, sugar, sweets, chocolate, snacks, chips, ice cream	gcakeslf+gcakeshf+gsugar+gsweet+gchoc+gsnacks+gicecrlf+gicecrhf
Jam	Marmelade, honey, jam, puree	gmarm
Sauce soups	Ketchup, sauces, soups	gketchup+ginsoup+gpowder
Beer	Beer, cider	gbeer
Spirits	Liquor	gspirits
Remainder miscellaneous	Vinegar, spices, soya products, algae, seaweed meal, nutritional powder and remainder (miscellaneous)	gspice+gsoya+gformula+ggarb

Note. Paleolithic and non-paleolithic food groups aggregated from Malmö Diet and Cancer Study (MDCS) food groups. Non-energy containing beverages excluded. Paleolithic and non-Paleolithic food group names consistent with previous publications on PDF.
