# Supplementary material for: Inverse association between Paleolithic Diet Fraction and mortality and incidence of cardiometabolic disease in the prospective Malmö Diet and Cancer Study
Source: Eur J Nutr. 2023 Dec 11;63(2):501–12. doi: 10.1007/s00394-023-03279-6 (PMC10899283; doi:10.1007/s00394-023-03279-6)
Supplement: Supplementary file 3 — Supplementary file3 (PDF 187 KB) [file 394_2023_3279_MOESM3_ESM.pdf]

Supplemental Table S2

Median Daily Micronutrient Intake at Baseline

Variable		*(IQR, Min-Max)*	Quintile of PDF 1	2	3	4	5	P^a	r_s	P^{ab}	$r_s^{\,b}$
Protein											
g	80	(28, 17-264)	86	83	82	78	73	<.001	-0.21		
MJ	1.3	(0.5, 0.3-4.4)	1.4	1.4	1.4	1.3	1.2	<.001	-0.21		
E%	15	(3, 6-29)	14	14	15	15	15	<.001	0.19		
Fat											
g	89	(42, 9-479)	102	96	91	84	76	<.001	-0.28		
MJ	3.3	(1.6, 0.3-18.0)	3.8	3.6	3.4	3.2	2.9	<.001	-0.28		
E%	37	(8, 4-70)	38	38	37	37	36	<.001	-0.08		
Carbohydrates											
g	232	(91, 33-966)	267	248	235	219	197	<.001	-0.35		
MJ	3.9	(1.5, 0.6-16.2)	4.5	4.1	3.9	3.7	3.3	<.001	-0.35		
E%	43	(8, 16-76)	44	43	43	43	42	<.001	-0.10		
Fibre											
g	19	(8, 3-119)	17	18	19	19	20	<.001	0.11		
MJ	0.2	(0.1, 0.0-1.0)	0.1	0.2	0.2	0.2	0.2	<.001	0.11		
E%	1.7	(0.6, 0.4-6.7)	1.4	1.6	1.7	1.8	2.1	<.001	0.44		
Alcohol											
g	7	(14, 0-176)	6	7	7	8	9	<.001	0.07		
MJ	0.2	(0.4, 0.0-5.2)	0.2	0.2	0.2	0.2	0.3	<.001	0.07		
E%	2	(4, 0-42)	2	2	2	3	3	<.001	0.13		
Retinol, mg	1.0	(0.8, 0.0-38.7)	1.2	1.1	1.0	0.9	0.7	<.001	-0.27	<.001	-0.14
Beta-carotene, mg	2.6	(2.9, 0.0-71.0)	1.8	2.3	2.7	2.9	3.5	<.001	0.27	<.001	0.39
Retinol equivalents, mg	1.5	(1.1, 0.1-39.3)	1.6	1.6	1.5	1.5	1.5	<.001	-0.06	<.001	0.12
Vitamin D, ug	6.8	(3.9, 0.3-39.8)	7.5	7.1	7.0	6.5	5.9	<.001	-0.19	.049	0.01
Vitamin E, mg	9.5	(4.2, 0.9-66.4)	9.3	9.6	9.8	9.5	9.6	<.001	0.03	<.001	0.33
D-alpha-tocopherol, mg	8.5	(3.7, 0.9-64.9)	8.2	8.5	8.7	8.5	8.7	<.001	0.05	<.001	0.37
Ascorbic acid, mg	94	(73, 0-883)	79	86	94	97	108	<.001	0.18	<.001	0.37
Thiamin, mg	1.3	(0.5, 0.3-4.7)	1.4	1.4	1.3	1.2	1.1	<.001	-0.29	.1	-0.01
Riboflavin, mg	1.8	(0.7, 0.2-8.8)	2.1	1.9	1.8	1.7	1.5	<.001	-0.32	<.001	-0.11
Folate, ug	230	(94, 32-1086)	229	231	231	229	229	.3	-0.01	<.001	0.36
Niacin, mg	17	(6, 5-62)	17	17	17	17	17	<.001	-0.03	<.001	0.33
Niacin equivalents, mg	34	(12, 8-122)	35	35	34	33	32	<.001	-0.12	<.001	0.27
B6 (Pyridoxine), mg	1.9	(0.7, 0.4-11.5)	1.8	1.9	1.9	1.9	1.9	<.001	0.03	<.001	0.50
B12 (Cobalamin), mg	5.4	(3.3, 0.1-120.7)	5.8	5.5	5.5	5.2	4.9	<.001	-0.13	<.001	0.07
Calcium, mg	1070	(500, 78-5227)	1281	1155	1090	998	878	<.001	-0.36	<.001	-0.17
Phosphorus, mg	1470	(552, 191-4517)	1660	1552	1497	1405	1274	<.001	-0.31	<.001	-0.03
Iron, mg	14	(6, 3-64)	16	15	15	14	13	<.001	-0.18	<.001	0.11
Magnesium, mg	331	(111, 82-1304)	354	339	335	323	307	<.001	-0.18	<.001	0.26
Sodium, mg	3160	(1217, 644-17638)	3337	3258	3216	3079	2926	<.001	-0.16	<.001	0.17
Potassium, mg	3244	(1075, 798-12590)	3250	3233	3284	3235	3215	.1	-0.01	<.001	0.58
Zinc, mg	11	(4, 2-35)	11	11	11	10	10	<.001	-0.20	<.001	0.13
Selenium, ug	35	(16, 6-150)	35	35	36	35	35	.5	0.00	<.001	0.31
Saturated fat (SFA), g	38	(20, 2-178)	45	41	38	35	31	<.001	-0.31	<.001	-0.10
Monounsaturated fat (MUFA), g	31	(14, 1-192)	35	33	32	29	27	<.001	-0.26	.2	-0.01
Polyunsaturated fat (PUFA), g	14	(7, 2-81)	15	15	14	13	12	<.001	-0.18	<.001	0.05
Cholesterol, g	0.33	(0.17, 0.00-1.73)	0.35	0.34	0.34	0.33	0.31	<.001	-0.09	<.001	0.16
Monosaccharides, g	36	(20, 2-275)	36	36	37	37	36	.01	0.02	<.001	0.26
Disaccharides, g	65	(37, 3-462)	87	73	66	58	47	<.001	-0.48	<.001	-0.39
Sucrose, g	42	(29, 1-400)	54	46	42	39	33	<.001	-0.32	<.001	-0.18
Butyric acid, Caproic acid, Caprylic acid, Capric acid, C4:0-C10:0, g	3.2	(2.6, 0.0-26.2)	4.0	3.5	3.2	2.9	2.5	<.001	-0.27	<.001	-0.14
Lauric acid, C12:0, g	2.2	(1.4, 0.0-12.7)	2.7	2.5	2.3	2.1	1.7	<.001	-0.32	<.001	-0.15
Myristic acid, C14:0, g	4.0	(3, 0-25)	4.9	4.4	4.1	3.7	3.3	<.001	-0.29	<.001	-0.12
Palmitic acid, C16:0, g	18	(9, 1-89)	21	20	18	17	15	<.001	-0.29	<.001	-0.07
Stearic acid, C18:0, g	8.4	(4.2, 0.2-51.8)	9.7	9.1	8.6	7.9	7.0	<.001	-0.31	<.001	-0.08
Arachidic acid, C20:0, g	0.38	(0.19, 0.00-2.14)	0.42	0.40	0.39	0.36	0.32	<.001	-0.22	.7	0.00
Palmitoleic acid, C16:1, g	1.8	(0.8, 0.1-9.6)	1.9	1.8	1.8	1.7	1.6	<.001	-0.21	<.001	0.03
Oleic acid, C18:1, g	28	(13, 1-177)	31	30	28	26	24	<.001	-0.26	.01	-0.02
Alpha-linolenic acid (n-3), gamma-linolenic acid (n-6), C18:3, g	1.7	(0.9, 0.2-11.8)	1.9	1.8	1.7	1.6	1.4	<.001	-0.22	.4	-0.01
Arachidonic acid (n-6), C20:4, g	0.14	(0.08, 0.00-1.09)	0.14	0.14	0.15	0.14	0.14	<.001	-0.05	<.001	0.18
Linoleic acid (n-6), C18:2, g	11	(6, 1-70)	12	12	11	10	9	<.001	-0.19	<.001	0.02
Eicosapentaenoic acid (n-3), C20:5, g	0.12	(0.17, 0.00-2.67)	0.08	0.11	0.12	0.13	0.14	<.001	0.14	<.001	0.23
Docosapentaenoic acid (n-3), C22:5, g	0.06	(0.05, 0.00-0.81)	0.06	0.06	0.06	0.06	0.06	<.001	0.04	<.001	0.20
Docosahexaenoic acid (n-3), C22:6, g	0.25	(0.31, 0.00-4.41)	0.21	0.24	0.25	0.26	0.28	<.001	0.11	<.001	0.22

Note. Participants without prevalent coronary event, stroke or diabetes at baseline examination 1992-1996 (N = 24,104) from the Malmö Diet and Cancer Study. PDF = Paleolithic Diet Fraction.

[a] Spearman rank test with quintile of PDF as predictor. [b] Relative to total food group weight.
